# Low Prevalence of HSV-1 and *Helicobacter pylori* in HNSCC and Chronic Tonsillitis Patients Compared to Healthy Individuals

**DOI:** 10.3390/diagnostics13101798

**Published:** 2023-05-19

**Authors:** Joanna Katarzyna Strzelczyk, Agata Świętek, Dorota Hudy, Karolina Gołąbek, Jadwiga Gaździcka, Katarzyna Miśkiewicz-Orczyk, Wojciech Ścierski, Janusz Strzelczyk, Maciej Misiołek

**Affiliations:** 1 Department of Medical and Molecular Biology, Faculty of Medical Sciences in Zabrze, Medical University of Silesia in Katowice, 19 Jordana St., 41-808 Zabrze, Poland; 2 Silesia LabMed Research and Implementation Center, Medical University of Silesia in Katowice, 19 Jordana St., 41-808 Zabrze, Poland; 3 Department of Otorhinolaryngology and Oncological Laryngology, Faculty of Medical Sciences in Zabrze, Medical University of Silesia in Katowice, 10 C Skłodowskiej St., 41-800 Zabrze, Poland; 4 Department of Endocrinology and Neuroendocrine Tumors, Department of Pathophysiology and Endocrinology, Faculty of Medical Sciences in Zabrze, Medical University of Silesia in Katowice, 35 Ceglana St., 40-514 Katowice, Poland

**Keywords:** HSV-1/2, *H. pylori*, HNSCC, chronic tonsillitis, PCR

## Abstract

Recent studies identified viral and bacterial factors, including HSV-1 and *H. pylori*, as possible factors associated with diseases such as chronic tonsillitis and cancers, including head and neck squamous cell carcinoma (HNSCC). We assessed the prevalence of HSV-1/2 and *H. pylori* in patients with HNSCC, chronic tonsillitis, and healthy individuals using PCR after DNA isolation. Associations were sought between the presence of HSV-1, *H. pylori,* and clinicopathological and demographic characteristics and stimulant use. HSV-1 and *H. pylori* were most frequently identified in controls (HSV-1: 12.5% and *H. pylori*: 6.3%). There were 7 (7.8%) and 8 (8.6%) patients with positive HSV-1 in HNSCC and chronic tonsillitis patients, respectively, while the prevalence of *H. pylori* was 0/90 (0%) and 3/93 (3.2%), respectively. More cases of HSV-1 were observed in older individuals in the control group. All positive HSV-1 cases in the HNSCC group were associated with advanced tumor stage (T3/T4). The prevalence of HSV-1 and *H. pylori* was highest in the controls compared to HNSCC and chronic tonsillitis patients, which indicates that the pathogens were not risk factors. However, since all positive HSV-1 cases in the HNSCC group were observed only in patients with advanced tumor stage, we suggested a possible link between HSV-1 and tumor progression. Further follow-up of the study groups is planned.

## 1. Introduction

*Helicobacter pylori* is a Gram-negative, spiral-shaped bacterium that colonizes the gastric and duodenal mucosa. By developing special mechanisms such as motility and urease secretion, it can survive in unfavorable living conditions [1]. There are two routes of infection, i.e., oral-oral and oral-fecal. The main risk factors include poor housing, economic conditions, and genetic and racial predisposition. *H. pylori* infection has been recognized as an infectious disease regardless of the onset of symptoms. While most infected individuals remain asymptomatic, *H. pylori* has been proven to be associated with such infections. It usually causes chronic bacterial infection and is mainly related to gastritis, peptic ulcers, gastrointestinal disorders, and gastric cancer [2].

Most individuals with *H. pylori* infection are diagnosed in developing countries (80–95%) [3], while in European countries, the infection rate is 20–40% [4]. Of note, about 4.4 billion people worldwide were infected in 2015 [5]. According to the epidemiological data from a multicenter study conducted in Poland in 2002 and 2003, *H. pylori* infection affected 84% of adults [6]. In the diagnosis of *H. pylori* infection, invasive methods are used. They involve the analysis of gastric mucosal samples taken during endoscopy and non-invasive methods (serological tests detecting specific IgG class antibodies, the C^13^ urea breath test, and the stool antigen test) [7]. Diverse PCR methods targeting specific *H. pylori* genes are also known. Detection by PCR is possible using various types of biological material [8].

Herpes simplex virus 1 (HSV-1) is a double-stranded DNA virus transmitted by saliva and through direct contact with mucous membranes and skin. It is usually associated with herpes (cold sores) of the lips and mouth [9]. Additionally, the virus can cause other less common diseases, such as keratitis and encephalitis, especially in immunocompromised individuals [10]. HSV-1 remains permanently in the body in a dormant state in ganglion cells and can be periodically reactivated [11]. Herpes virus infections are widespread among humans. Serologic studies indicate that most adults (up to 95%) are infected [12,13]. Moreover, this infection may be asymptomatic, contributing to the high prevalence of HSV-1 infections worldwide [14].

The most common malignancy involving the head and neck region is squamous cell carcinoma (HNSCC), which develops from epithelial cells of the mucosa of the mouth, pharynx, and larynx [15]. The risk factors for the development of typical HNSCC are well defined and mainly include exposure to tobacco and high-proof alcohol. HNSCC ranks 12th among all types of cancers [16,17]. The etiology of HNSCC consists of many factors, including different infections [9,16]. Several studies have shown positive associations of various pathogens (HSV-1) with oral cancer and precancerous conditions [9,18]. As regards *H. pylori,* the link between oral cancer and infection is still not determined, as reported by Kuo et al., 2022 [19]. Research in recent years has demonstrated the role of human papillomavirus (HPV) infection, which has the ability to infect human epithelial cells and is the etiologic agent of many types of cancer, including HNSCC. The prevalence varies due to cultural differences, medical practice, and diagnostic procedures [20,21].

Tonsils are part of Waldeyer’s ring, the first line of defense of the mucosa against invading pathogens. One of the most common indications for tonsillectomy is chronic tonsillitis, usually characterized by a sore throat that lasts longer than three months and is accompanied by tonsillar inflammation. Similarly, in the pediatric autoimmune neuropsychiatric disorders associated with streptococcal infections (PANDAS), chronic streptococcal infection increases not only the risk of chronic tonsillitis but also obsessive-compulsive disorders, mainly in children [22]. The relationship between *H. pylori* infection and the pathogenesis of chronic tonsillitis remains controversial. Some researchers showed that the tonsil tissue could be a reservoir of *H. pylori* [23,24]. They also found it responsible for gastric reinfection, while other authors did not observe such an association [25,26,27]. To date, there have been limited data on HSV-1 infections in chronic tonsillitis samples [28].

The etiology of HNSCC and chronic tonsillitis is complex and involves many factors, including the presence of pathogens [9,16]. Both HSV and *H. pylori* are common human pathogens. There is a growing level of interest in their potential role in the initiation and progression of cancer [1,4,9,14]. HSV and *H. pylori* infections are distributed worldwide with variations in prevalence. Thorough knowledge of bacterial or viral infections is needed to understand their impact on the etiology of the diseases fully. We examined the prevalence of these pathogens to investigate the potential role of HSV and *H. pylori* in HNSCC and chronic tonsillitis and to provide further data about the epidemiology of HSV and *H. pylori* infections in adolescents in Poland.

The aim of this study was to determine the prevalence of HSV-1/2 and *H. pylori* in three groups, i.e., in patients with HNSCC, chronic tonsillitis, and healthy individuals. The aim was also to define the relationship between the presence of the pathogens and clinicopathological and demographic data and the use of stimulants in the study population.

## 2. Materials and Methods

The present study included 295 volunteers that were Caucasians and lived in Poland. The specimens were collected from healthy individuals (*n* = 112) and patients diagnosed with HNSCC (*n* = 90) and chronic tonsillitis (*n* = 93). Cancer tissue samples were collected postoperatively. The diagnosis of HNSCC was confirmed histologically. Tonsillar tissues were collected after tonsillectomy from patients diagnosed with chronic tonsillitis and were verified by a histopathologist. The samples were collected at the Department of Otorhinolaryngology and Oncological Laryngology in Zabrze, Medical University of Silesia in Katowice.

This retrospective study was carried out with surgical interventions from May 2018 to May 2022. Swab samples from the oral, buccal mucosa were taken from healthy subjects during the same period using cotton swabs. Immediately after collection, the samples from all participants were labeled and stored at −80 °C until further analysis. The exclusion criteria for all groups were age below 18 years and no written informed consent to participate in the study, while in the case of HNSCC, an undiagnosed primary tumor or preoperative radio- and/or chemotherapy, local or nodal recurrence, and the second primary tumor of HNSCC. As regards control and chronic tonsillitis groups, any diagnosed cancer, history of cancer treatment (radiotherapy, chemotherapy), and confirmed or clinically suspected immunodeficiency were the exclusion criteria. Additionally, the presence of any chronic diseases was also an excluding factor in the control group.

The study was conducted in compliance with the Declaration of Helsinki and approved by the Institutional Review Board (IRB No. KNW/0022/KB1/49/16 and No. KNW/0022/KB1/49/II/16/17). All patients and volunteers provided written informed consent before participating in the study.

### 2.1. Patient Characteristics

Patient demographic data, including age, gender, and baseline data, such as smoking and/or alcohol consumption, were obtained using a self-administered questionnaire. The data are given in Table 1. HNSCC samples were characterized according to the TNM classification in line with the criteria of the American Joint Committee on Cancer (AJCC) 8th edition and the International Union Against Cancer (UICC) [29]. Clinical data of the HNSCC group, including T classification (T), lymph node status (N), and histological grade (G), are summarized in Table 2. The groups were matched in terms of the number of participants and sociodemographic characteristics, except age which was higher in the HNSCC group.

### 2.2. Methods

#### 2.2.1. DNA Preparation

Prior to the DNA isolation, tumor and tonsil tissue samples were homogenized in FastPrep^®^-24 (MP Biomedicals, Irvin, CA, USA) with ceramic beads (Lysing Matrix A, MP Biomedicals, Irvin, CA, USA). DNA was isolated from the samples using the Gene Matrix Tissue DNA Purification Kit (Eurx, Gdańsk, Poland). Oral mucosal epithelial cells of the control group were extracted using the GeneMATRIX Swab-Extract DNA Purification Kit (Eurx, Gdańsk, Poland). Spectrophotometry was used to qualitatively and quantitatively evaluate the isolated DNA material (NanoPhotometer Pearl, Implen, Munich, Germany).

#### 2.2.2. Detection of HSV-1/2 and *H. pylori*

The real-time PCR method was used to detect the presence of the genetic material of the pathogens in the samples. The presence of HSV-1/2 and *H. pylori* in the specimens from HNSCC, chronic tonsillitis patients, and healthy controls was determined using the commercially available HSV-1/2 PCR kit (HSV/ISEX/100, GeneProof, Brno, Czech Republic) and the AmpliSens *Helicobacter pylori*-FRT PCR kit (R-B9 (RG,iQ)-CE, Ecoli Dx, s.r.o., Prague, Czech Republic). The tests were performed according to the manufacturer’s instructions using 10 µL of isolated DNA. PCRs were performed using the QuantStudio 5 instrument (Thermo Fisher Scientific, Waltham, MA, USA). Negative, positive, and amplification controls were performed in each test. The positive control was the DNA of *H. pylori* or HSV-1/2. DNA buffer was used as a negative control in both tests. Positive control STI-88 from the *H. pylori* kit was the control of amplification for the *H. pylori* test. The internal standard served as an amplification control for the HSV-1/2 test. The positive criteria for pathogen detection were the correct results for all controls and the CT value for the pathogen ≤ the boundary value specified in the kit. The sensitivity of the *H. pylori* kit was 1 × 10^3^ *H. pylori* genome equivalents per ml, and the diagnostic sensitivity of the HSV-1/2 kit was described as 100% (CI_95%_: 97.08–100%).

#### 2.2.3. Statistical Analysis

Fisher’s exact test was used to determine the differences between the groups. Age differences were checked using Shapiro—Wilk and Mann—Whitney U tests. The level of significant differences was set at *p* < 0.05. The data were presented as quantity or the median with the range. All statistical analyses were performed using STATISTICA 13.3 (StatSoft. Inc., Tulsa, OK, USA).

## 3. Results

There was a difference in age between the HNSSC group and the control or chronic tonsillitis group (control vs. chronic tonsillitis *p* > 0.05; control vs. HNSCC *p* = 0.007; chronic tonsillitis vs. HNSCC *p* < 0.001).

### The Prevalence of HSV-1/2 and H. pylori in All Study Groups

**Figure 1:**
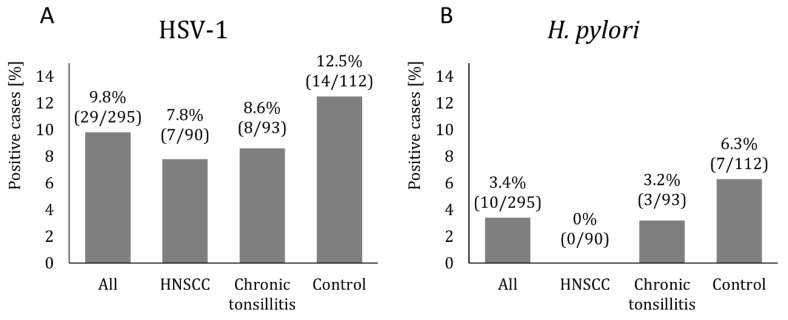
Absolute and percent prevalence of HSV-1 (**A**) or *H. pylori* (**B**) in patients with HNSCC, chronic tonsillitis, and controls.

HSV-1 and *H. pylori* were most frequently identified in controls (HSV-1: 12.5% and *H. pylori*: 6.3%). There were seven (7.8%) and eight (8.6%) subjects with positive HSV-1 among HNSCC and chronic tonsillitis patients, respectively (Figure 1A). Positive HSV-2 was not found in the study groups. The prevalence of *H. pylori* in patients with HNSCC and those with chronic tonsillitis was 0% and 3.2%, respectively (Figure 1B). There were statistical differences in the prevalence of *H. pylori* between the control and HNSCC groups (*p* = 0.035). No other differences were found between the groups regarding the prevalence of HSV-1 or *H. pylori* (*p* > 0.05), as shown in Table 3.

There were no differences between the prevalence of HSV-1 and *H. pylori* and age, gender, drinking and/or smoking status (*p* > 0.05) in all groups except for the control group and the presence of HSV-1. In the control group, there were more positive cases of HSV-1 in older individuals (≥40 years of age) (*p* = 0.042, Table 4).

All positive HSV-1 cases in the HNSCC group were associated with advanced tumor stage (T3/T4, *p* = 0.048, Table 5). Six of seven cases in the HNSCC group were patients with T4 classification (*p* = 0.005, Table 5). All cases of HSV-1 were observed in stage IV, which was significant (*p* = 0.044, Table 5). There were no associations between histological grade and nodal status and the occurrence of HSV-1 (Table S1). Additionally, no difference was found in all groups between the prevalence of HSV-1 and *H. pylori* and smoking and regular or occasional drinking (*p* > 0.05, Table S2).

## 4. Discussion

HSV-1 has been identified as a risk factor for the development of some human malignancies, particularly in association with stimulants such as tobacco and alcohol. Several studies have confirmed a possible correlation between herpes virus infection and head and neck cancers [11,30,31]. HSV-1 has the potential to block programmed cell death and can produce factors that can induce apoptosis in immune cells, such as T cells, B cells, macrophages, and dendritic cells [32,33]. Studies suggested that the presence of HSV-1 was associated with impaired DNA damage repair and amplification of pre-existing oncogenes in tumor tissues [34,35]. Other studies confirmed that HSV-1 could affect the inhibition of apoptosis of infected cells via ICP-0, Us3, and Us5 proteins. It affects the impaired regulation of cellular apoptotic pathways such as p53. As a result, cell death does not occur until HSV-1 infection develops at a high viral copy number [36,37,38]. Interestingly, several studies have demonstrated that HSV-1 has the ability to reactivate in critically ill patients with COVID-19 [39]. In studies on different materials obtained from patients (bronchoalveolar lavage fluid [BALF], tracheal aspirate, and blood), HSV-1 was confirmed in an average of 40% of samples. This supports the theory that some viruses can cause reactivation of other viruses. It was speculated that systemic or pulmonary reactivation of HSV-1 could be related to immune system impairment caused by SARS-CoV-2 [39].

The literature data show the prevalence of HSV in HNSCC ranges from 5% to 25% [40,41]. Moreover, the infection was mostly asymptomatic in patients with HNSCC [42]. A Polish study using PCR showed the presence of the HSV-1 genome in 7.5% of tumor tissue samples from 80 patients with oral squamous cell carcinoma (OSCC), which is also in line with our study (7.8%; 8/90). The authors emphasized the need for further studies on a larger cohort to determine the potential role of HSV-1 as an infection cofactor in HNSCC [43]. Similarly, a study on the Sudanese population reported the prevalence of HSV-1 at 7.5% in OSCC tissue samples [44]. Low rates of HSV-1 infection (1.92–4.8%) were reported in other studies [41,45]. As in our study, several previous reports also failed to confirm a significant association between HSV-1 and the risk of HNSCC [31,46,47]. A population-based study by Starr et al. [11] reported that the presence of HSV-1 was associated with a slightly increased risk of OSCC [11]. Another study suggested that HSV-1 could play an important role in OSCC, as they showed statistical significance in the mandibular location (18.6% positive cases) [48]. In a case report of a patient with HSV-1-positive laryngeal squamous cell carcinoma (LSCC), changes were observed in cell nuclei and cytoplasmic tails between cells, which may indicate a potential link between the herpes virus and the occurrence of LSCC [49]. The question arises as to whether the herpes virus can initiate cancer or whether HSV-1 infection can be superimposed due to an immunocompromised state in cancer patients. Interestingly, higher levels of IgM antibodies to HSV-1 were observed in the serum of oral cancer patients compared to control subjects of the same age [50]. Similarly, another study found that IgG levels were elevated in cancer patients compared to healthy individuals [9]. It was hypothesized that the elevated level of antibodies to HSV-1 in patients with OSCC could be related to the oncogenic potential of HSV [51]. Current treatments involving pharmacotherapy, chemotherapy, radiation therapy, or surgery can cause many adverse effects. Accordingly, the use of immunotherapy with oncolytic viruses has recently become the subject of extensive research. Native or genetically recombinant viruses have the ability to selectively infect cancer cells and cause their disintegration without affecting normal cells. Because of its wide host range and large genome, HSV-1 is used in the destruction of cancer cells. After entering the host cell, HSV-1 replicates in the nucleus and can induce programmed cell death, pyroptosis, necrosis, and autophagy [52]. Further research focusing on efficacy, safety, and high cytotoxicity is warranted.

The prevalence of HSV-1 was highest in the control group, which indicates that HSV-1 was not a risk factor in our HNSCC group, although all positive HSV-1 cases were found only in patients with advanced tumor stage (T3/T4). It suggests that HSV-1 can modulate cancer progression. The effects of specific molecular mechanisms of HSV-1 on tumors are not fully explained, but some studies showed contribution to tumor progression. The search of medical databases (Pubmed, Medline) did not show other reports indicating the associations between HSV-1 and tumor stage in HNSCC patients. However, a study on brain glioma reported the presence of HSV-1 only at a higher tumor stage (WHO lV), which aligns with our findings [53]. The presence of the virus was absent at lower stages. In a study of central nervous system tumors, a hypothesis was proposed that HSV-1/2 could be involved in modulating the retinoblastoma suppressor pathway and, therefore, could play a role in oncogenesis [54]. In addition, it was suggested that HSV-1/2 could affect various intracellular signaling pathways, which implies oncogenic changes in cells and could also exacerbate the already existing cancerous changes [55]. Another study reported the importance of viral miRNA, which can interfere with the expression of infected miRNA, thereby affecting the formation and development of tumors. Additionally, there is a theory that HSV 1/2 may facilitate tumor progression through miRNA modulation of tumors [56].

To the best of our knowledge, there are only a few single studies describing HSV detection in tonsillar tissue samples [57,58,59]. HSV was not detected by in situ hybridization (ISH) in any tonsil samples [57]. Using PCR, another study showed 7.4% positive tonsils samples for HSV-1 and none for HSV-2 [58]. Another research assessed tissue samples taken from the core of the tonsils in children and adults with recurrent tonsillitis and tonsillar hypertrophy, and 1.8% (1/56) was positive for HSV [59]. In our study, we detected HSV-1 in 8.6% of patients with chronic tonsillitis (8/93). No significant differences were found between patients with chronic tonsillitis and controls. Therefore, our results confirm the previous findings of other researchers. It appears that HSV infection is not a factor associated with tonsillitis. Of note, recurrent inflammatory infections, particularly viral, have an impact on tonsillar hypertrophy and increase the risk of obstructive sleep apnea (OSA) [60,61]. Continuous positive airway pressure (cPAP) is the most common treatment of sleep apnea syndrome, especially in moderate and severe diseases [62,63,64]. Additionally, nasal surgery, pharyngeal surgery, and/or maxillomandibular surgery are effective methods of OSA treatment [65,66,67].

A study analyzing 182 papers reported that the global prevalence of oral HSV-1 was 63.6% in 2016 and was highest in the South-East Asia Region. Detection of HSV was performed using type-specific immunoglobulin G antibodies. Furthermore, the infection of oral HSV-1 was associated with age [68]. Another meta-analysis showed that the seroprevalence of HSV-1 in the healthy adult European population was 73.6% and increased with age. The highest seroprevalence was found in Eastern Europe (87.9%), while the lowest (57.7%) was observed in Northern Europe [69]. The estimates of the prevalence of HSV-2 in 2016 showed that 13.2% of individuals aged 15–49 years had immunoglobulin G antibodies for HSV-2, and the highest prevalence was observed in Africa and increased with age and gender [68]. In our study, we reported that 12.5% of controls were HSV-1 positive, while none of the controls was HSV-2 positive. In another Polish study, the level of anti-HSV IgG antibodies in controls was 6% [70]. The prevalence of HSV infection varies greatly depending on the continent, country, population, or HSV type. It was found that the detection of HSV-1 was significantly higher than HSV-2 (87–35% for HSV-1 compared to 17–8% for HSV-2) [71,72,73,74,75,76,77,78,79]. Furthermore, in our study, the presence of the HSV-1 virus was associated with higher age. Similar results were shown in other studies where the prevalence of HSV-1 infection increased with the age of patients [73,74]. Our results could confirm previous observations on the relationship between viral infection and the age of patients. A higher prevalence of the virus in older patients could be associated with immunosenescence [80].

Of note, the obtained results may be related to research methods. The serological tests may confirm the prevalence of HSV-1 and HSV-2. However, they did not indicate the site of infection, such as the mouth or genital area [81]. Moreover, oral HSV shedding had an influence on the positive results of PCR detection [14,82]. Some studies analyzed oral HSV shedding using PCR from swab samples collected daily from HSV-seropositive patients. The presence of HSV in oral mucosa was confirmed by PCR only on 3.7–26% of overall days [14,83,84,85]. Because viral shedding is episodic, increasing the sampling frequency on overall days increased the detection rate of the virus by PCR [85]. Moreover, the rate of HSV detection was various and was not associated with lesions or prodrome [14].

In our study, we did not show the presence of HSV-2. Similarly, some studies found no oral HSV-2 in controls [82,86]. It may be associated with the differences at the site of reactivation between HSV-1 and HSV-2. HSV-1 is mostly reactivated from trigeminal ganglia compared to HSV-2, which is reactivated from lumbosacral ganglia [87]. Moreover, the possibility of detecting oral asymptomatic HSV-1 infection was 7.5 times higher than oral HSV-2 infection [82].

As in gastric cancer, it was speculated that *H. pylori* could be involved in modifying the host immune response, which contributes to the progression of oral and oropharyngeal cancer, as confirmed by several studies [16,88,89]. Both indirect (inflammation) and direct (induction of protein modulation and genetic mutations) effects of *H. pylori* on gastric epithelial cell function were demonstrated. However, other tissues may also be affected [90]. Many studies confirmed the presence of *H. pylori* in the middle ear, oral cavity, larynx, saliva, nasal and sinus mucosa, and pharyngeal lymphoid tissue [91,92,93,94,95]. The study results suggest that these locations were not only potential reservoirs for microbial infection and gastric reinfection but also provided preliminary information on the correlation of *H. pylori* infection with the development of HNSCC [96].

In our study, we did not confirm the presence of *H. pylori* in HNSCC patients (0/90 samples). Previous studies have reported that the prevalence of *H. pylori* in HNSCC ranged from 0–40%. Based on the meta-analysis of 11 studies, it was concluded that PCR was the best technique for detecting *H. pylori* in laryngeal or pharyngeal tissue, which was also used in our study due to its high specificity and sensitivity of almost 100% [97]. The significantly higher percentage of positive results for *H. pylori* was described in cases of laryngeal and pharyngeal cancer [16,96,98,99,100]. Several recent studies using quantitative polymerase chain reaction (qPCR) and IHC have not confirmed the presence of *H. pylori* in patients with LSCC or HNSCC [88,101,102,103,104,105]. In a Polish study by Burduk [106] on laryngeal squamous cell carcinoma (LSCC) samples using the urease test, a high percentage of *H. pylori* was confirmed (62.5%; 50/80) [106]. Another study by Burduk [107] showed that the *H. pylori* cagA gene was observed in 46.7% and 49.3% of laryngeal cancer samples, depending on tissue location. In addition, an association was found between the presence of cagA and clinical stage T3 and T4, which may indicate *H. pylori* as a potential carcinogen [107]. Other studies based on the same material also showed positive results for more than 75% of samples [98,108,109,110,111]. It could be explained by the association of *H. pylori* infection with the occurrence of laryngopharyngeal or gastric reflux and the colonization of the laryngeal region and the pharynx [112]. As a result, epithelial cells are damaged, and the subsequent inflammatory process can lead to chronic damage and the subsequent development and progression of cancer [113].

Some studies suggest the association between *H. pylori* and upper respiratory tract diseases, such as tonsillitis, rhinitis, and rhinosinusitis [114,115]. *H. pylori* is likely to colonize the tonsillar surface and core tissues [116]. In our study, we detected *H. pylori* only in 3.2% of patients with chronic tonsillitis. The role of *H. pylori* in the pathogenesis of chronic tonsillitis is still unclear. The meta-analysis based on six studies [26,98,117,118,119,120], including 462 patients from different populations, showed that *H. pylori* had a significant effect on chronic tonsillitis compared with non-infectious indications for tonsillectomy (e.g., obstruction, hypertrophy). Additionally, *H. pylori* played a role in chronic tonsillitis in children but not in adults [121]. The method of detecting the pathogen also influenced the results [121]. It should be noted that the rapid urease test could lead to higher rates of false-positive results because other urease-producing species (except *H. pylori*) are present in the oral cavity as opposed to the stomach tissue [122]. 

In our study, the rate of *H. pylori* infection in healthy individuals was 6.3%, the highest in all study groups. Other studies based on the adult healthy Polish population showed the infection rate ranging from 39% to 84.19% [6,123,124]. Studies showed that the prevalence of *H. pylori* in healthy populations was different depending on the country. In the European population, the rate of infection was very wide. In the Czech Republic, 12% to 23.5% [111,125], while higher rates were reported in Hungary (32%) and the Netherlands (30%) [126,127]. Spain was the country with the highest prevalence of *H. pylori* (87.2%) [128]. According to one review, the prevalence of *H. pylori* was lower in Northern and Western Europe, while the highest infection rate was observed in Southern and Eastern Europe [129]. In Asia, it ranged from 18% to 35% in the Chinese population [121,130], 40% in Japan [131], 41% in the United Arab Emirates [3], and 62% in Kazakhstan [132]. Additionally, the infection rate was 65% in Brazil [133], while the prevalence of *H. pylori* was 13% in Canada [134].

In our study, we found no association between the presence of *H. pylori* and the selected parameters, such as age, gender, smoking, and drinking. Some studies found that the risk of infection was strongly associated with age [3,125,127,128,130,133], while other studies did not find such a relationship [130,134]. Moreover, the association between infection and gender was reported by Lorenzo et al. [128] (men had a higher risk of infection) [128]. However, other studies did not confirm it [125,132,133]. In addition, it was reported that the risk of *H. pylori* infection was associated with increased salt intake [132], sharing a bedroom with three or more persons [3,133], cigarette smoking, consumption of high-proof alcohol or not washing hands after coming back home [6], higher BMI [128], lower education level [133] or rural place of birth [6,127,130].

The differences in the results may be connected with a different detection method: the C-urea breath test, [125,130,131], IHC [121], the stool antigen test [3], the serological test [126,127,128] and real-time PCR [111]. In addition, the detection of *H. pylori* from the samples obtained from the oral cavity by different PCR methods may be difficult due to the presence of other 700 bacterial species and requires appropriate and specific primers [135,136]. Furthermore, ethnicity may influence susceptibility to *H. pylori* infection [133,134]. Willems et al. (2020) observed differences between the ethnic groups in Canada. Caucasians had the lowest infection rate (8%), while African, Asian, and South American populations had infection rates of 25%, 30%, and 34%, respectively, which suggested that race could be associated with susceptibility to infection [134].

### Limitations of the Study

The main limitation of the study is the difference in age between the HNSCC group and control/chronic tonsillitis groups, as shown in the Section 3. This study is also limited by a small number of samples. Therefore, to confirm the associations more accurately, future studies should be conducted on larger cohorts. Furthermore, considering viral and bacterial infections is needed to understand their impact on cancer progression fully. In the future, our studies will focus on the analysis of disease-free and overall survival in HNSCC patients.

## 5. Conclusions

To conclude, the prevalence of HSV-1 and *H. pylori* was highest in the control group compared to HNSCC and chronic tonsillitis, which indicates that the pathogens are not risk factors in the study groups. However, since all positive HSV-1 cases in the HNSCC group were found only in patients with advanced tumor stage, we suggest a possible link between HSV-1 and tumor progression. The knowledge of epidemiology is of great importance to public health. Given the prevalence of HSV-1 and *H. pylori* and the risk of severe disease, attention should be paid to screening, diagnosis, and prevention of infection, including the need to develop widely available rapid methods for effective detection. We plan to monitor the health status of our study groups and perform a long-term follow-up of their condition.

## Figures and Tables

**Table 1:** Distribution of sociodemographic characteristics in patients with HNSCC, chronic tonsillitis, and controls.

	All		HNSCC		Chronic Tonsillitis		Control	
	Median	Range	Median	Range	Median	Range	Median	Range
Age	41	29–57.5	60	52–65	33	24–41	33	24–49.25
	*N*	*%*	*N*	*%*	*N*	*%*	*N*	*%*
Age ≥ 40	153	51.86	84	93.33	26	27.96	43	38.39
Age < 40	142	48.14	6	6.67	67	72.04	69	61.61
Female	140	47.46	26	28.89	51	54.84	63	56.25
Male	155	52.54	64	71.11	42	45.16	49	43.75
Smoking	109	36.95	65	72.22	14	15.05	31	27.68
Not smoking	186	63.05	25	27.78	79	84.95	81	72.32
Drinking	182	61.69	61	67.78	29	31.18	92	82.14
Regular drinking	22	7.46	11	12.22	3	3.23	8	7.14
Occasional drinking	160	54.24	50	55.56	26	27.96	84	75.00
Not drinking	113	38.31	29	32.22	64	68.82	20	17.86
Smoking and drinking	85	28.81	50	55.56	8	8.60	27	24.11
Drinking; not smoking	96	32.54	11	12.22	20	21.51	65	58.04
Smoking; not drinking	26	8.81	15	16.67	7	7.53	4	3.57
Not smoking; not drinking	88	29.83	14	15.56	58	62.37	16	14.29

**Table 2:** Clinical characteristics of patients with HNSCC.

		*N*	(%)
Clinical T-classification		90	(100.00)
	T1	10	11.1
	T2	22	24.4
	T3	28	31.1
	T4	30	33.3
	T1 + T2	32	35.6
	T3 + T4	58	64.4
Lymph node status		90	(100.00)
	N0	43	47.8
	N1	22	24.4
	N2	22	24.4
	N3	3	3.3
	N1 + N2	44	48.8
Histological grade		90	(100.00)
	G1	15	16.7
	G2	56	62.2
	G3	19	21.1
	G1 + G2	71	78.9
Stage		90	(100.00)
	I	7	7.78
	II	12	13.33
	III	21	23.33
	IV	50	55.56

**Table 3:** Statistical significance of the ratio between the groups with HSV-1 or *H. pylori* occurrence.

	HSV-1	*H. pylori*
Control vs. HNSCC	0.283	**0.035**
Control vs. Chronic tonsillitis	0.376	0.353
Chronic tonsillitis vs. HNSCC	0.841	0.099

Bold and color indicate statistical significances with *p* < 0.05.

**Table 4:** Sociodemographic characteristics (age, gender, drinking and smoking status) and their association with HSV-1 and *H. pylori* in patients with HNSCC, chronic tonsillitis, and controls.

Study Group	Variable	Variant	HSV-1		*p*	*H. pylori*		*p*
			Negative	Positive		Negative	Positive	
HNSCC	Age	≥40	77	7	1	84	0	1
		<40	6	0		6	0	
	Gender	Female	23	3	0.407	26	0	1
		Male	60	4		64	0	
	Drinking	Yes	55	6	0.422	61	0	1
		No	28	1		29	0	
	Smoking	Yes	59	6	0.668	65	0	1
		No	24	1		25	0	
Chronic tonsillitis	Age	≥40	24	2	1	24	2	0.188
		<40	61	6		66	1	
	Gender	Female	47	4	1	49	2	1
		Male	38	4		39	1	
	Drinking	Yes	27	2	1	27	2	0.228
		No	58	6		63	1	
	Smoking	Yes	11	3	0.097	14	0	1
		No	74	5		76	3	
Control	Age	≥40	34	9	**0.042**	40	3	1
		<40	64	5		65	4	
	Gender	Female	57	6	0.389	61	2	0.237
		Male	41	8		44	5	
	Drinking	Yes	82	10	0.272	86	6	1
		No	16	4		19	1	
	Smoking	Yes	26	5	0.527	29	2	1
		No	72	9		76	5	

Bold and color indicate statistical significances with *p* < 0.05.

**Table 5:** The prevalence of HSV-1 and *H. pylori* in the HNSCC group according to T classification and stage.

Study Group	Variable	Variant	HSV-1		*p*	*H. pylori*		*p*
			Negative	Positive		Negative	Positive	
HNSCC	T1	Yes	10	0	1	10	0	1
		No	73	7		80	0	
	T2	Yes	22	0	0.188	22	0	1
		No	61	7		68	0	
	T3	Yes	27	1	0.428	28	0	1
		No	56	6		62	0	
	T4	Yes	24	6	**0.005**	30	0	1
		No	59	1		60	0	
	T1 + T2	Yes	32	0	**0.048**	32	0	1
		No	51	7		58	0	
	T3 + T4	Yes	51	7	**0.048**	58	0	1
		No	51	0		32	0	
	Stage I	Yes	7	0	1	7	0	1
		No	76	7		83	0	
	Stage II	Yes	12	0	0.588	12	0	1
		No	71	7		78	0	
	Stage III	Yes	21	0	0.193	90	0	1
		No	62	7		69	0	
	Stage IV	Yes	50	7	**0.044**	50	0	1
		No	33	0		40	0	

Bold and color indicate statistical significances with *p* < 0.05.

## Data Availability

The data used to support the findings of this research are available upon request.

## Supplementary Materials

The following supporting information can be downloaded at: https://www.mdpi.com/article/10.3390/diagnostics13101798/s1, Table S1 The prevalence of HSV-1 and *H. pylori* in the HNSCC group according to nodal status and histological, Table S2 The prevalence of HSV-1 or *H. pylori* and the sociodemographic characteristics (age, gender, drinking, and smoking status) in patients with HNSCC, chronic tonsillitis, and controls. grade.
