# Supplementary material for: Low Prevalence of HSV-1 and Helicobacter pylori in HNSCC and Chronic Tonsillitis Patients Compared to Healthy Individuals
Source: Diagnostics (Basel). 2023 May 19;13(10):1798. doi: 10.3390/diagnostics13101798 (PMC10217135; doi:10.3390/diagnostics13101798)
Supplement: Supplementary file 1 [file diagnostics-13-01798-s001.zip › diagnostics-2394163-supplementary.pdf]

Table S1. The prevalence of HSV-1 and *H. pylori* in the HNSCC group according to nodal status and histological grade

Study group	Variable	Variant	HSV-1		p	*H. pylori*		p
			negative	positive		negative	positive	
HNSCC	N0	Yes	41	2	0.438	43	0	1
		No	42	5		47	0	
	N1	Yes	19	3	0.355	22	0	1
		No	64	4		68	0	
	N2	Yes	20	2	1	22	0	1
		No	63	5		68	0	
	N3	Yes	3	0	1	3	0	1
		No	80	7		87	0	
	N1+N2	Yes	39	5	0.261	44	0	1
		No	44	2		46	0	
	G1	Yes	15	0	0.596	16	0	1
		No	68	7		74	0	
	G2	Yes	50	6	0.247	58	0	1
		No	33	1		32	0	
	G3	Yes	18	1	1	22	0	1
		No	65	6		68	0	
	G1+G2	Yes	65	6	1	71	0	1
		No	18	1		19	0	

Table S2. The prevalence of HSV-1 or *H. pylori* and the sociodemographic characteristics (age, gender, drinking and smoking status) in patients with HNSCC, chronic tonsillitis and controls

Study group	Variable	Variant	HSV-1 negative	HSV-1 positive	p	*H. pylori* negative	*H. pylori* positive	p
HNSCC	Drinking	Yes	55	6	0.422	61	0	1
		No	28	1		29	0	
	Smoking	Yes	59	6	0.668	65	0	1
		No	24	1		25	0	
	Occasional drinking	Yes	44	6	0.127	50	0	1
		No	39	1		40	0	
	Regular drinking	Yes	11	0	0.591	11	0	1
		No	72	7		79	0	
Chronic tonsillitis	Drinking	Yes	27	2	1	27	2	0.228
		No	58	6		63	1	
	Smoking	Yes	11	3	0.097	14	0	1
		No	74	5		76	3	
	Occasional drinking	Yes	24	2	1	24	2	0.188
		No	62	5		66	1	
	Regular drinking	Yes	3	0	1	3	0	1
		No	83	7		87	3	
Control	Drinking	Yes	82	10	0.272	86	6	1
		No	16	4		19	1	
	Smoking	Yes	26	5	0.527	29	2	1
		No	72	9		76	5	
	Occasional drinking	Yes	75	9	0.334	79	5	1
		No	23	5		26	2	
	Regular drinking	Yes	7	1	1	7	1	0.414
		No	91	13		98	6	
